# Identification and analysis of common bean (*Phaseolus vulgaris *L.) transcriptomes by massively parallel pyrosequencing

**DOI:** 10.1186/1471-2229-11-135

**Published:** 2011-10-11

**Authors:** Venu Kalavacharla, Zhanji Liu, Blake C Meyers, Jyothi Thimmapuram, Kalpalatha Melmaiee

**Affiliations:** 1 College of Agriculture & Related Sciences, Delaware State University, Dover, DE 19901, USA; 2 Department of Plant & Soil Sciences and Delaware Biotechnology Institute, University of Delaware, Newark, DE 19711, USA; 3 W. M. Keck Center for Comparative and Functional Genomics, University of Illinois, Urbana-Champaign, IL 61801, USA; 4 Center for Integrated Biological and Environmental Research, Delaware State University, Dover, DE 19901, USA

## Abstract

**Background:**

Common bean (*Phaseolus vulgaris*) is the most important food legume in the world. Although this crop is very important to both the developed and developing world as a means of dietary protein supply, resources available in common bean are limited. Global transcriptome analysis is important to better understand gene expression, genetic variation, and gene structure annotation in addition to other important features. However, the number and description of common bean sequences are very limited, which greatly inhibits genome and transcriptome research. Here we used 454 pyrosequencing to obtain a substantial transcriptome dataset for common bean.

**Results:**

We obtained 1,692,972 reads with an average read length of 207 nucleotides (nt). These reads were assembled into 59,295 unigenes including 39,572 contigs and 19,723 singletons, in addition to 35,328 singletons less than 100 bp. Comparing the unigenes to common bean ESTs deposited in GenBank, we found that 53.40% or 31,664 of these unigenes had no matches to this dataset and can be considered as new common bean transcripts. Functional annotation of the unigenes carried out by Gene Ontology assignments from hits to *Arabidopsis *and soybean indicated coverage of a broad range of GO categories. The common bean unigenes were also compared to the bean bacterial artificial chromosome (BAC) end sequences, and a total of 21% of the unigenes (12,724) including 9,199 contigs and 3,256 singletons match to the 8,823 BAC-end sequences. In addition, a large number of simple sequence repeats (SSRs) and transcription factors were also identified in this study.

**Conclusions:**

This work provides the first large scale identification of the common bean transcriptome derived by 454 pyrosequencing. This research has resulted in a 150% increase in the number of *Phaseolus vulgaris *ESTs. The dataset obtained through this analysis will provide a platform for functional genomics in common bean and related legumes and will aid in the development of molecular markers that can be used for tagging genes of interest. Additionally, these sequences will provide a means for better annotation of the on-going common bean whole genome sequencing.

## Background

*Phaseolus vulgaris *or common bean is the most important edible food legume in the world. It provides 15% of the protein and 30% of the caloric requirement to the world's population, and represents 50% of the grain legumes consumed worldwide [1]. Common bean has several market classes, which include dry beans, canned beans, and green beans. The related legume soybean (*Glycine max*), which is one of the most important sources of seed protein and oil content belongs to the same group of papilionoid legumes as common bean. Common bean and soybean diverged nearly 20 million years ago around the time of the major duplication event in soybean [2,3]. Synteny analysis indicates that most segments of any one common bean linkage group are highly similar to two soybean chromosomes [4]. Since *P. vulgaris *is a true diploid with a genome size estimated to be between 588 and 637 mega base pairs (Mbp) [5-7], it will serve as a model for understanding the ~1,100 million base pairs (Mbp) soybean genome [1]. Common bean is also related to other members of the papilionid legumes including cowpea (*Vigna unguiculata*) and pigeon pea (*Vigna radiata*). Therefore, better knowledge of the common bean genome will facilitate better understanding of other important legumes as well as the development of comparative genomics resources.

The common bean genome is currently being sequenced [8]. When the sequencing of the genome is complete, this will require the prediction, annotation and validation of the expressed genes in common bean. The availability of large sets of annotated sequences as derived by identification, sequencing, and validation of genes expressed in the common bean will help in the development of an accurate and complete structural annotation of the common bean genome, a valid transcriptome map, and the identification of the genetic basis of agriculturally important traits in common bean. The transcriptome sequences will also help in the identification of transcription factors and small RNAs in common bean, understanding of gene families, and very importantly the development of molecular markers for common bean.

To date there are several relevant and important publications in common bean transcriptome sequencing and bioinformatics analyses. Ramirez et al. [9] sequenced 21,026 ESTs from various cDNA libraries (nitrogen-fixing root nodules, phosphorus-deficient roots, developing pods, and leaves) derived from the Meso-American common bean genotype Negro Jamapa 81, and leaves from the Andean genotype G19833. Approximately 10,000 of these identified ESTs were classified into 2,226 contigs and 7,969 singletons.

Melotto et al. [10] constructed three cDNA libraries from the common bean breeding line SEL1308. These libraries were comprised of 19-day old trifoliate leaves, 10-day old shoots, and 13-day old shoots (inoculated with *Colletotrichum lindemuthianum*). Of the 5,255 single-pass sequences obtained from this work, trimming and clustering helped identify 3,126 unigenes, and of these only 314 unigenes showed similarity to sequences from the existing database.

Tian et al. [11] constructed a suppression substractive cDNA library to identify genes involved in response to phosphorous starvation. Six-day old seedlings from the genotype G19833 were exposed to high and low phosphorus (five and 1,000 μmol/L) respectively and the poly (A+) RNA derived from total shoot and root RNA from plants in these conditions was used for construction of the libraries. After dot-blot hybridization and identification of differentially expressed clones, full-length cDNAs were identified from cDNA libraries constructed from the low and high P exposure experiments. Differentially expressed genes were characterized into five functional groups, and these authors were able to further classify 72 genes by comparison to the GenBank non-redundant database using BLASTx values less than 1.0 × 1e^-2^).

Thibivilliers et al. [7] identified 6, 202 new common bean ESTs (out of a total of 10,221 ESTs) by using a substractive cDNA library constructed from the common bean rust resistant-cultivar Early Gallatin. This cultivar was inoculated with races 49 (avirulent on genotypes such as Early Gallatin carrying the rust resistance locus *Ur-4*) and 41 (a virulent race that is not recognized by *Ur-4)*. In order to identify genes which are differentially expressed, suppression substractive expression experiments were carried out to identify sequences which were up-regulated in response to susceptible and resistant host-pathogen interactions.

Despite these studies in common bean, there is still a paucity in the number of common bean ESTs and genes that have been deposited in GenBank (~83,448 ESTs, as of September, 2010) compared to other legume and plant models. Therefore, there is a need for deeper coverage and EST sequences from diverse common bean tissues and genotypes.

There has been an evolution in sequencing technologies starting with the traditional dideoxynucleotide sequencing to capillary-based sequencing to current "next-generation" sequencing [12,13]. The emergence of next-generation sequencing technologies has substantially helped advance plant genome research, particularly for non-model plant species [14]. Next generation sequencing strategies typically have the ability to generate millions of reads of sequences at a time, without the need for cloning of the fragment libraries; these are faster than traditional capillary-based methods which may be limited to 96 samples in a run and require the nucleic acid material (DNA or complementary DNA; cDNA) to be cloned into a plasmid and amplified by *Escherichia coli *(*E. coli*). Therefore, cloning bias that is typically present in genome sequencing projects can be avoided, although depending on the specific platform used for next generation sequencing, there may be other specific biases involved. An advantage of some next generation sequencing technologies is that information on genome organization and layout may not be necessary *a priori*. The Roche 454 method uses the pyrophosphate molecule released when nucleotides are incorporated by DNA polymerase into the growing DNA chain to fuel reactions that result in the detection of light resulting from cleavage of oxyluciferin by luciferase [15]. Using an emulsion PCR approach, it has the ability to sequence 400 to 500 nucleotides of paired ends and produces approximately 400-600 Mbp per run. This method has been applied to genome [16] and transcriptome [17-19] sequencing due to its high throughput, coverage, and savings in cost.

In *A. thaliana*, pyrosequencing has been tested successfully to verify whether this technology is able to provide an unbiased representation of transcripts as compared to the sequenced genome. Using messenger RNA (mRNA) derived from *Arabidopsis *seedlings, Weber and colleagues [20] identified 541,852 ESTs which accounted for nearly 17,449 gene loci and thus provided very deep coverage of the transcriptome. The analysis also revealed that all regions of the mRNA transcript were equally represented therefore removing issues of bias, and very importantly, over 16,000 of the ESTs identified in this research were novel and did not exist in the existing EST database. Therefore, these researchers concluded that the pyrosequencing platform has the ability to aid in gene discovery and expression analysis for non-model plants, and could be used for both genomic and transcriptomic analysis.

In the legume *Medicago truncatula*, the 454 technology has been used to generate 252,384 reads with average (cleaned) read length of 92 nucleotides [16], with a total of 184,599 unique sequences generated after clustering and assembly. Gene ontology (GO) assignments from matches to the completed *Arabidopsis *sequence showed a broad coverage of the GO categories. Cheung and colleagues [17] were also able to map 70,026 reads generated in this research to 785 *Medicago *BAC sequences. In their analysis of the maize shoot apical meristem, Emrich and colleagues [16] discovered 261,000 ESTs, annotated more than 25,000 maize genomic sequences, and identified ~400 maize transcripts for which homologs have not been identified in any other species. The value of this approach in novel gene/EST discovery is underlined by the fact that nearly 30% of the ESTs identified in this study did not match the ~648,000 maize ESTs in the databases. Velasco and colleagues [21] generated a draft genome of grape, *Vitis vinifera *Pinot Noir by using a combination of Sanger sequencing and 454 sequencing. They identified approximately 29,585 predicted genes of which 96.1% could be assigned to genetic linkage groups (LGs). Many of the genes identified have potential implications on grapevine cultivation including those that influence wine quality, and response to pathogens. Detailed analysis was also carried out to identify sequences related to disease resistance, phenolic and terpenoid pathways, transcription factors, repetitive elements, and non-coding RNAs (including microRNAs, transfer RNAs, small nuclear RNAs, ribosomal RNAs and small nucleolar RNAs).

Sequences obtained in common bean by deep sequencing can be mapped onto common bean maps by using syntenic relationships between common bean and soybean; these two species diverged over 19 MYA. McClean et al. [22] determined syntenic relationships between common bean and soybean by taking genetically positioned transcript loci and mapping to the soybean 1.01 pseudochromosome assembly. Since prior evidence has shown that almost every common bean locus maps to two soybean locations (recent diploidy and polyploidy respectively), and a genome assembly is not yet available in common bean, this synteny can be effectively utilized. Therefore, by referencing common bean loci with unknown physical map positions (in common bean) to syntenic regions in soybean, and then referencing back to the common bean genetic map, approximate locations of common bean transcript loci were determined. Using this method, the authors [22] were able to determine median physical-to-genetic distance ratio in common bean to be ~120 Kb/cM (based on the soybean physical distance derived from the pseudochromosome assembly). This allowed the placing of ~15,000 EST contigs and singletons on the common bean map, and this strategy will allow for the discovery and chromosomal locations of genes controlling important traits in both common bean and soybean. Therefore, until the common bean genome is completed, we can now use synteny with soybean to determine more accurate locations of common bean transcripts.

## Results and Discussion

### Generation of ESTs from *Phaseolus vulgaris*

Since the combined total number of common bean ESTs that have been deposited in Genbank (as of September 2010) is ~83,000, we sought to increase the diversity and number of these sequences to be useful for functional genomics and molecular breeding studies. We generated cDNA libraries from four plant tissues: leaves, flowers, roots derived from the common bean cultivar "Sierra", and pods derived from the common bean breeding line "BAT93." Even though the genotype that was chosen for the common bean genome sequencing project is G19833, there is considerable value in generating transcriptomic sequences from these additional genotypes. Sierra is a common bean cultivar released by Michigan State University with improved disease resistance, competitive yield, and upright growth habit. Additionally, disease resistance in Sierra includes rust resistance, field tolerance to white mold, and resistance to *Fusarium *wilt [23]. The breeding line BAT93 is one of the parents of the core common bean mapping populations, and therefore, understanding and identification of sequences expressed in the developing pod is very useful. BAT93 also carries resistances to multiple diseases. The sequence data obtained from this work will also be very useful in identifying single nucleotide polymorphism (SNP) loci when compared to sequences derived from other genotypes in the work by Ramirez et al. [9], Melotto et al. [10] and Thibivilliers et al. [7].

The use of next-generation sequencing for transcriptome and genome studies has been well documented (as discussed in background). Given the paucity of available common bean sequences and our interest in generating sequence reads long enough to be useful for the design of primers for mapping onto the common bean map, we chose the Roche 454 sequencing method (see materials and methods). cDNAs derived from the RNA of the four tissues were tagged with sequence tags that would help identify tissue of origin after sequencing and assembly of data. After normalization, library construction and sequencing, sequences were assembled and annotated (see materials and methods) resulting in the generation of ~1.6 million reads, with an average length of 207 nucleotides (nt) and a total length of 350 Mbp derived from three bulk 454 runs. These reads were assembled using gsAssembler (Newbler, from Roche, http://www.roche-applied-science.com), into 39,572 contigs and 55,051 singletons. Of these singletons, 35,328 were determined to be less than 100 nucleotides (nt). Therefore, sequences derived from this study serve as an important first step to deriving a larger transcriptomic set of sequences in common bean and additionally demonstrate the value of next-generation sequencing. Further, these common bean sequences will be important for discovery of orthologous genes in other so-called "orphan legumes" [24]. Assembly statistics for the 454 reads are shown in Table 1. Of the 1.6 million reads, we were able to assemble 75% of the reads. The average length of contigs was 473 nt and for singletons 103 nt (Table 2). For the purposes of this work, we consider the 39,572 contigs and 19,723 singletons which are longer than 100 nt collectively as unigenes (totalling 59, 295). The number of contigs and singletons with respective sizes are shown in Table 2. The largest number of contigs (11,597) was in the 200-299 nt range, followed by 9,696 contigs in the 100-199 nt range. There were 5,438 contigs which were > 1,000 nt. The longest contig length was 3,183 nt.

**Table 1:** Assembly statistics of common bean 454 reads

Name	**No**.
Total reads	1,692,972
Reads fully assembled	1,280,774
Reads partially assembled	245,452
Repeats	53,136
Outliers	58,559
Contigs	39,572
Singletons	55,051
Singletons above 100 bp	19,723
Unigenes (contigs + singletons above 100 nt)	59,295

**Table 2:** Sequence length distribution of assembled contigs and singletons

Nucleotide length (nt)	Contigs	Singletons
< 100	19	35,328
100-199	9,496	5,064
200-299	11,597	14,639
300-399	3,376	20
400-499	2,451	-
500-599	1,808	-
600-699	1,489	-
700-799	1,329	-
800-899	1,294	-
900-999	1,275	-
> 1000	5,438	-
Total	39,572	55,051
Maximum length	3,183 nt	-
Average length	473	103

In order to determine the number of reads which make up any particular contig in the assembly, we determined the number of reads versus number of contigs (Table 3). In our unigenes sequences, 22,723 contigs were comprised of 2-10 reads (minimum read range).

**Table 3:** Summary of component reads per contig.

Number of reads	Number of contigs
2-10	22,723
11-20	3,920
21-30	2,087
31-40	1,526
41-50	1,137
51-100	3,332
101-150	1,435
151-200	715
> 200	1,999

### Comparative analysis with existing *Phaseolus vulgaris *ESTs

Most of the common bean ESTs available in GenBank are derived from genotypes such as Early Gallatin, Bat 93, Negro Jamapa 81, and G19833 [7]. In order to identify new *P. vulgaris *sequences among the 454 unigene set that we generated, a BLASTn search (e-value < 1e^-10^) against the common bean ESTs in GenBank was carried out and revealed that 27,631 (46.60%) of the 454 unigenes matched known ESTs. Thus 31,664 unigenes (18,087 contigs and 13,577 singletons; 53.40%) can be considered as new *P. vulgaris *unigenes.

The 83,947 common bean EST sequences (as of October 1, 2010) can be assembled into about 20,000 unique sequences. These new sequences significantly enrich by approximately 150% the number of transcripts of this important legume and provide a significant resource for discovering new genes, developing molecular markers for future genetic linkage and QTL analysis, and comparative studies with other legumes, and will help in the discovery and understanding of genes underlying agriculturally important traits in common bean.

### Comparison with common bean BAC-end sequences

Recently, a BAC library for common bean genotype G19833 was constructed [25], and a draft FingerPrinted Contig (FPC) physical map has been released using the BAC-end sequences from this work (Genbank EI415689-EI504705). This data set contains 89,017 BAC-end sequences. The FPC physical map makes it possible to map some 454 unigenes into the bean physical map. All the 454 unigenes were compared to the BAC-end sequences by BLASTN (e-value < 1e^-10^) according to McClean et al [22]. As a result, a total of 12,725 unigenes including 9,199 contigs and 3,256 singletons (21% of the unigenes), were mapped to the available 8,823 BAC-end sequences.

### Functional annotation of the *P. vulgaris *unigenes-Comparison to *Arabidopsis*

**Figure 1:**
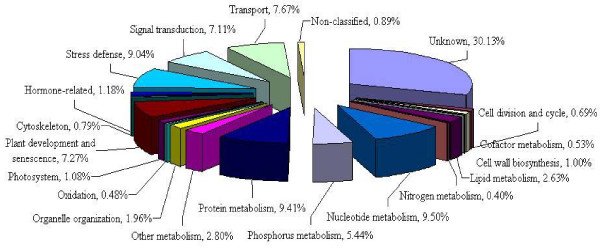
**Functional classification of *P. vulgaris *unigenes according to the *Arabidopsis *peptide sequences**.

The common bean unigene set was compared to predicted *Arabidopsis *protein sequences by using BLASTX. A total of 26,622 (44.90%) of the unigenes had a significant match with the annotated *Arabidopsis *proteins, and were assigned putative functions (Figure 1). However, 55.10% (32,673) of the common bean unigenes had no significant match and therefore could not be classified into gene ontology (GO) categories. The comparison of the distribution of *P. vulgaris *unigenes among GO molecular function groups with that of *A. thaliana *suggests that this 454 unigene set is broadly representative of the *P. vulgaris *transcriptome. Unigenes with positive matches to the *Arabidopsis *proteins were grouped into 20 categories (Figure 1). The largest proportion of the functionally assigned unigenes fell into seven categories: unknown (30.13%), nucleotide metabolism (9.50%), protein metabolism (9.41%), plant development and senescence (7.27%), stress defense (9.04%), signal transduction (7.11%) and transport (7.67%).

#### Functional comparison to soybean

**Figure 2:**
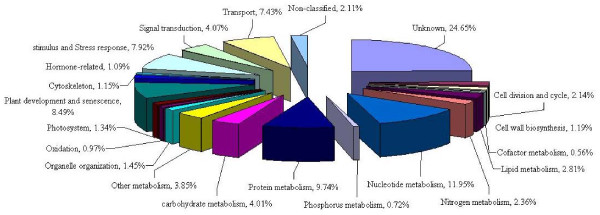
**Functional classification of *P. vulgaris *unigenes according to the soybean peptide sequences**.

All of the common bean unigenes were used to compare with soybean peptide sequences (55,787) by BLASTX (Figure 2). As a result, a total of 63.31% (37,538) unigenes have a good match to soybean peptide sequences. Therefore the number of common bean matches to soybean sequences was significantly higher (~1.4×) compared to *Arabidopsis *and may reflect the larger number of predicted genes in soybean compared to *Arabidopsis*. These sequences can be used for discovery of not only common bean genes but also for validation of predicted soybean genes.

### Comparison of *P. vulgaris *unigenes with those in *M. truncatula*, *G. max*, *L japonicus, A. thaliana *and *O. sativa*

**Figure 3:**
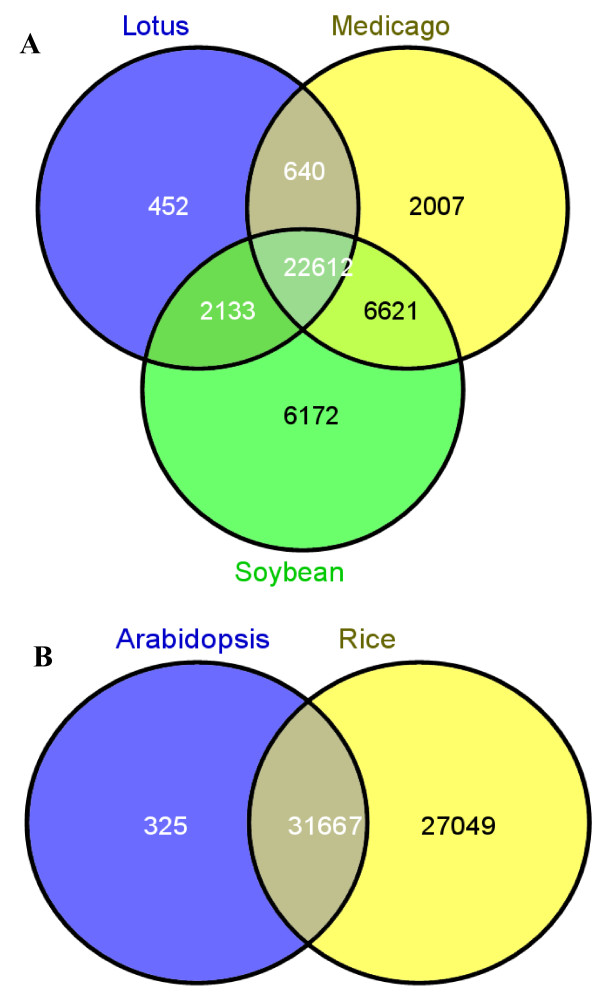
**Venn diagram of *P. vulgaris *unigenes showing common and unique unigenes compared to legume and non-legume species**. (A) *P. vulgaris *unigenes compared to soybean, *Medicago *and lotus. (B) *P. vulgaris *unigenes compared to *Arabidopsis *and rice. Numbers in the Venn diagram refer to the number of *P. vulgaris *unigenes having hits to each plant species, as labeled.

We were also interested in understanding the relationship of common bean unigenes in this study to those that have been identified in other legume models and the model plants *Arabidopsis *and rice with larger sequence collections. We also wanted to determine the unique and shared sequences between common bean, *Medicago*, lotus and soybean, and also those that are shared between common bean, *Arabidopsis *and rice. Nearly 54% (31,880) of the common bean unigenes have homology to *Medicago*, 44% (25,837) have homology to lotus, and 63% (37,538) have homology to soybean (Figure 3A). Approximately 72% (42,270) of common bean unigenes are shared between the four legume species (common bean, lotus, *Medicago *and soybean). We also determined that 54% (31,992) of the common bean unigenes are shared with *Arabidopsis *and 99% (58,716) are shared with rice. When compared to *Medicago*, soybean and lotus, 28% (16,525) of the unigenes are unique to common bean whereas only 0.43% (254) of the unigenes are unique to common bean when compared to *Arabidopsis *and rice (Figure 3B).

As seen in the comparison to the *Arabidopsis *transcriptome, the most abundant category was comprised of 30.13% of the unigenes with unknown functions which was consistent with the previous study by Thibivilliers *et al*. [7], who found that 31.9% of common bean ESTs from bean rust-infected plants had an unknown function. They also found that 15.3% of those ESTs fell into signal transduction and nucleotide metabolism classes. Similarly, our results found that 16.61% of 454 unigenes belonged to signal transduction and nucleotide metabolism. Additionally, this analysis showed that 9.04% of the unigenes belong to the stress defense category. These unigenes provide a new and additional source for mining stress-regulated and defense response genes. Interestingly, Wong et al. [26] identified a common bean antimicrobial peptide with the ability to inhibit the human immunodeficiency virus (HIV)-1 reverse transcriptase. This 47-amino acid peptide was also found to inhibit fungi such as *Botrytis cinerea*, *Fusarium oxysporum *and *Mycosphaerella arachidicola*. We used the corresponding nucleotide sequence from this peptide to search against the 454 sequences in this report, and discovered one unigene represented by contig03541 with a nucleotide length of 450 bases. Search of this sequence against the NCBI non-redundant database identified homology to a plant defensin peptide from legumes such as mung bean, soybean, *Medicago*, and yam-bean (*Pachyrhizus erosus*), and it is possible that this is a gene that is specific to legumes.

#### Validation of common bean reference genes

**Figure 4:**
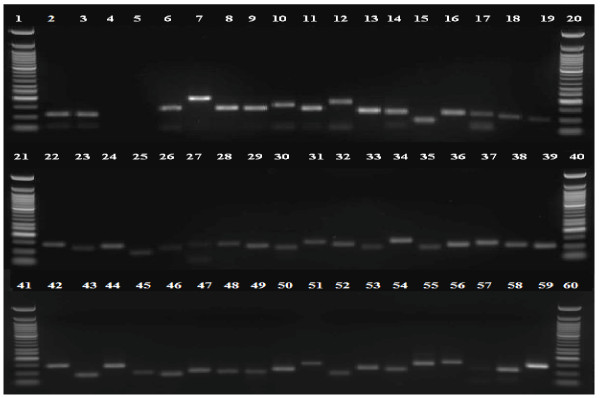
**Experimental validation of 48 common bean 454-sequencing derived unigenes by RT-PCR**. Lanes with 50 bp ladder are lanes 1, 20, 21, 40, 41, and 60; Confirmation of absence of DNA contamination is shown in lanes 2-5 where RT-PCR amplification was carried out with primers designed from contig11286 in lanes with genomic DNA, leaf cDNA, leaf cDNA control (no reverse transcriptase added to reaction), and water as template to check DNA contamination. In lanes 6-19, 22-39, and 42-56, 58 and 59 RT-PCR products derived by amplification from an additional 47 common bean unigenes using leaf cDNA as a template are shown (complete list of contigs shown in Table 4). Lane 57 is amplification by the *cons7 *primers.

Thibivilliers et al. [7] compared several housekeeping genes for use as a common bean reference for qRT-PCR experiments. They tested three bean genes *TC197 *(guanine nucleotide-binding protein beta subunit-like protein)*, TC127 *(ubiquitin), and *TC185 *(tubulin beta chain), and the common bean homologs of the soybean genes *cons6 *(coding for an F-box protein family)*, cons7 *(a metalloprotease), and *cons15 *(a peptidase S16). These researchers concluded that *cons7 *was the most stably expressed for their experimental conditions. Likewise, Libault et al. [27] also identified *cons7 *to be stably expressed and to be useful as a reference gene for quantitative studies in soybean, and with the confirmation in our studies can possibly be used for other legume gene expression experiments. Therefore, for our experiments, we used the *Gmcons7 *primers and verified expression in the Sierra genotype (please see Figure 4, lane 57); this was then used as an endogenous control, and used in leaf tissue as a reference gene for expression analysis of common bean contigs.

#### Quantification of tissue-specific expression of the common bean transcriptome

**Figure 5:**
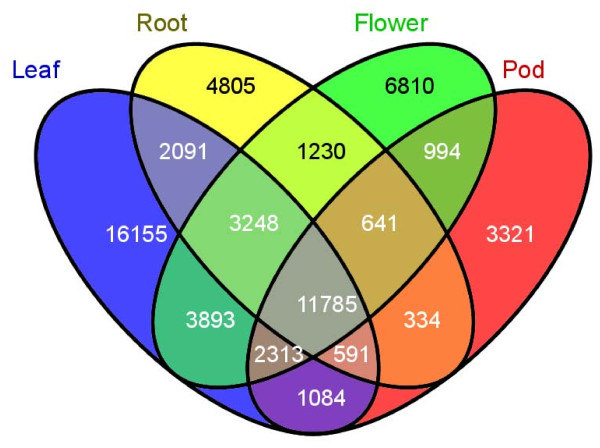
**Tissue-specific expression of common bean unigenes**. cDNA libraries were tagged during library construction; in the figure, blue represents transcripts present in leaves, yellow represents transcripts present in roots, green represents transcripts present in flower, and red represents transcripts present in pods.

When the cDNA libraries were created, the four tissues were tagged using a molecular barcode, based on their source of either leaves, roots, flowers or pods (see materials and methods) so that we could determine possible origin of tissues of the transcripts. The tags can be used to describe the presence or degree of tissue-specific expression of the unigenes. The distribution of these tags among the four tissues is shown in Figure 5. About 69% (41,161 unigenes) of the unigenes were present in leaves, 52% (30,914 unigenes) were present in flowers, 42% (24,725 unigenes) were present in roots, and 36% (21,063 unigenes) were present in pods. Among all the unigenes, 27% (16,155 unigenes) were observed only in leaves, 8% (4,805 unigenes) only in roots, 11% (6,810 unigenes) only in flowers, and 6% (3,321 unigenes) only in pods.

In our analysis of the 454 data, we found that 28,204 contigs were composed of transcripts that were derived from multiple tissues (Table 4). The tagging of the cDNA libraries will be very useful in order to verify and validate global gene expression patterns and understanding both shared and unique transcripts between and among the tissues in this study. Equally significant is the ability to capture rarely expressed transcripts. Since normalization was carried out (as seen in methods), the large number of transcripts derived from leaves is interesting. The contigs and singletons which contain flower, root, and pod-specific transcripts will be very useful to understand and compare with transcriptomic sequences derived from other temporal and spatial conditions from other studies.

**Table 4:** Identification of tissue-specific unigenes from common bean 454 sequences

Tissue-specific unigenes	No. of unigenes	Average reads	No. of reads in the largest contigs
Leaf-specific	16,155	1.99	96
Root-specific	4,805	2.21	502
Pod-specific	3,321	3.63	650
Flower-specific	6,810	1.87	231
Mixed-tissue unigenes	28,204	59.83	2,484
All unigenes	59,295	29.60	2,484

### SSR analysis

Simple sequence repeats (SSRs), or microsatellites consist of repeats of short nucleotide motifs with two to six base pairs in length. In the present study, the 59,295 454-derived sequences from common bean (estimated length of 22.93 Mbp) and 92,124 common bean genomic sequences (validated September 2010; estimated length of 64.67 Mbp) were analyzed for SSR sequences using the software MISA http://pgrc.ipk-gatersleben.de/misa. We surveyed these and all other sequences mentioned in this analysis for di-, tri-, tetra-, penta- and hexa-nucleotide type of SSRs. We detected a total of 1,516 and 4,517 SSRs in 454-derived and genomic sequences respectively (Table 5). In order to determine the identification of SSR sequences from other plants with both transcriptome and genomic resources, we analyzed 33,001 unigenes and 973.34 Mbp of genomic sequences from *G. max*, 18,098 unigenes and 105.5 Mbp of genomic sequences from *M. truncatula*, and 30,579 unigenes and the whole genome from *Arabidopsis*. In *G. max*, we found 3,548 SSRs in the unigenes, and 143,666 SSRs in genomic sequences. In *M. truncatula*, we found 1,470 SSRs in the unigenes, and 10,412 SSRs in the genomic sequences, and finally we found 5,586 SSRs in *Arabidopsis *unigenes, and 14,110 SSRs in *Arabidopsis *genomic sequences (Table 5).

**Table 5:** SSR survey in unigenes and genomic sequences from *P. vulgaris*, *G. max*, *M. truncatula*, and *A. thaliana*.

Type	*P. vulgaris*		*G. max*		*M. truncatula*		*A. thaliana*	
	
	Unigene	Genome	Unigene	Genome	Unigene	Genome	Unigene	Genome
Dinucleotide	548	3163	5944	99856	1903	6462	1914	8686
Trinucleotide	902	1213	5771	38411	2999	3532	3600	5180
Tetranucleotide	39	98	238	3954	165	314	34	155
Pentanucleotide	12	23	66	1161	53	63	8	38
Hexanucleotide	15	20	100	284	65	41	30	51
Total SSR	1516	4517	12119	143666	5158	10412	5586	14110
Total length (Mbp)	22.94	64.68	71.80	973.34	51.93	105.52	43.58	111.14
Average distance (kb)	15.13	14.32	5.92	6.78	10.07	10.13	7.80	7.88

We then analyzed the average distance between any two SSRs and found that this differed among species. The average distance between two SSRs in unigenes and genomic sequences of *P. vulgaris *was 15.13 kb and 14.32 kb respectively, higher than that of the other three species. However, the average distance between two SSRs was quite similar between unigenes and genomic sequences for common bean, soybean, *Medicago*, and lotus (Table 5).

The frequency of SSRs in terms of repeat motif length (di-, tri-, tetra-, penta-, and hexa- nucleotide) was different. Of all the SSRs found in common bean unigenes, dinucleotide, trinucleotide, tetranucleotide, pentanucleotide and hexanucleotide repeats account for 36.15%, 59.50%, 2.57%, 0.79%, and 0.99%, respectively, while repeats account for 70.02%, 26.85%, 2.17%, 0.51% and 0.44% in genomic sequences. In *G. max *unigenes, dinucleotide, trinucleotide, tetranucleotide, pentanucleotide and hexanucleotide repeats account for 42.64%, 54.20%, 2.00%, 0.51%, and 0.65%, respectively, and was 69.50%, 26.74%, 2.75%, 0.81% and 0.20% in genomic sequences. In *M. truncatula *unigenes, dinucleotide, trinucleotide, tetranucleotide, pentanucleotide and hexanucleotide repeats account for 35.03%, 59.66%, 3.33%, 1.16%, and 0.82%, respectively, and was 62.06%, 33.92%, 3.02%, 0.61% and 0.39% in genomic sequences. In *Arabidopsis *unigenes, dinucleotide, trinucleotide, tetranucleotide, pentanucleotide and hexanucleotide repeats account for 34.26%, 64.45%, 0.61%, 0.14%, and 0.54%, respectively, which was different from 61.56%, 36.71%, 1.10%, 0.27% and 0.36% in genomic sequences. The most frequent type of repeat motif between unigenes and genomic sequences was different. Trinucleotide SSRs were the most common type in unigenes in all the four species, while dinucleotide SSRs were the most common type in genomic sequences. These EST-SSRs will help to develop SSR markers with high polymorphism for common bean.

Tri-nucleotides were found to be the most abundant repeats and AAG/CTT repeats were the most frequent motifs in the tri-nucleotides. The prevalence of tri-nucleotide over di-nucleotide or other SSRs was also observed in the unigenes of *G. max*, *M. truncatula *and *A. thaliana*, and also may be characteristic of EST-SSRs of maize, wheat, rice, sorghum, barley [28] and many other plant species [29,30]. In contrast, di-nucleotides were the most common repeats in the genomic sequences of the four species and AT/AT was the most dominant repeat. Blair et al. [30,31] and Cordoba et al. [32] identified 184 gene-based SSRs and 875 SSRs from common bean ESTs and BAC-end sequences, respectively. They also found that tri-nucleotide SSRs were more common in ESTs, while di-nucleotide SSRs were more dominant in GSSs. The frequency of SSR-containing ESTs in the common bean unigenes as shown in this study was 2.37% and much lower than that of rice [28], bread wheat [33], and other plants [29]. The SSRs identified in the present study can be used by the common bean community as molecular markers for mapping of important agronomic traits and for integration of common bean genetic and physical maps.

### Validation of selected bean 454 transcripts

**Figure 6:**
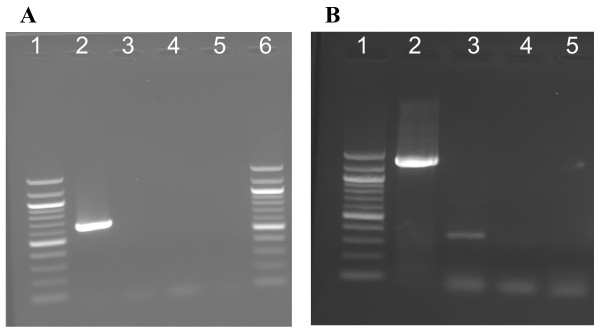
**Tests for DNA contamination in reverse transcriptase PCR**. (A) Common bean sequence characterized amplified repeat (SCAR) marker SK14, linked to the *Ur-3 *rust resistance locus. From our experiments, SK14 amplifies from genomic DNA but not from cDNA, presumably because SK14 is from the intronic region of the gene. Forward and reverse primers derived from the SK14 sequence were used to amplify a 600 bp product from genomic DNA and cDNA; no amplification from cDNA was observed. Lane 1, 100 bp ladder; Lane 2, genomic DNA; Lane 3, leaf cDNA; Lane 4. Negative cDNA control (no reverse transcriptase was added to cDNA synthesis reaction); Lane 5, H_2_O only control; Lane 6, 100 bp ladder. (B) Primers from contig32565, a sequence with homology to a MADS transcription factor amplified long flanking intronic genomic DNA yielding a 1200 bp amplicon from genomic DNA and a short 300 bp amplicon from cDNA. The order and contents of lanes 1 to 5 are identical to those in panel A.

We wanted to verify the expression of common bean ESTs identified in this work, before which we ensured that the procedures that we were following in the laboratory were consistent and that there was no contamination of the cDNA with genomic DNA. Figures 6A and 6B show that the cDNA that we have used for our gene expression experiments is contamination free. We wanted to test the accuracy of the contigs assembled by the gsAssembler with reverse transcriptase (RT)-PCR. We designed PCR primers for 48 randomly selected contigs (Table 6) and analyzed the cDNA under standard PCR conditions and electrophoresed the products on a 2% agarose gel (Figure 4).

**Table 6:** Description of unigenes randomly selected for validation

**Lane No**.	Unigene Name	Annotation
2	contig11286	MLO8 (MILDEW RESISTANCE LOCUS O 8); calmodulin binding [*Arabidopsis thaliana*]
3	contig11286	MLO8 (MILDEW RESISTANCE LOCUS O 8); calmodulin binding [*Arabidopsis thaliana*]
4	contig11286	MLO8 (MILDEW RESISTANCE LOCUS O 8); calmodulin binding [*Arabidopsis thaliana*]
5	contig11286	MLO8 (MILDEW RESISTANCE LOCUS O 8); calmodulin binding [*Arabidopsis thaliana*]
6	contig33974	MLO1 [*Lotus corniculatus *var. japonicus]
7	contig32923	ATMLO1/MLO1 (MILDEW RESISTANCE LOCUS O 1); calmodulin binding [*Arabidopsis thaliana*]
8	contig01942	resistance gene analog NBS1 [*Helianthus annuus*]
9	contig04562	R 10 protein [*Glycine max*]
10	contig05928	MLO1 [*Lotus corniculatus *var. japonicus]
11	contig28775	L6-like resistance gene
12	contig35803	Mlo-like resistance gene
13	contig36500	Hm1-like resistance gene
14	contig39371	N-like resistance gene
15	FFSTDNT01C34EJ	Fls2-like resistance gene
16	FGQI37401AS3FB	Pto-like kinase OG10 [*Phaseolus vulgaris*]
17	contig29749	Phaseolin
18	contig38383	fructokinase-like protein [*Cicer arietinum*]
19	contig04711	alcohol dehydrogenase [*Prunus armeniaca*]
22	contig20010	ABC transporter family protein [*Arabidopsis thaliana*]
23	contig14749	senescence-associated nodulin 1A [*Glycine max*]
24	contig28207	Late embryogenesis abundant protein Lea14-A, putative [*Ricinus communis*]
25	contig07734	phosphoenolpyruvate carboxylase kinase [*Glycine max*]
26	contig28742	fructokinase-like protein [*Cicer arietinum*]
27	contig33251	sucrose synthase [*Vigna angularis*]
28	contig38427	senescence-associated nodulin 1A [*Glycine max*]
29	contig28548	nodulin-26
30	contig08830	WRKY35 [*Glycine max*]
31	contig14217	NAC domain protein NAC1 [*Phaseolus vulgaris*]
32	contig32665	transcriptional factor NAC11 [*Glycine max*]
33	contig17174	dihydroflavonol-4-reductase 2 [*Glycine max*]
34	contig29672	glutathione S-transferase GST 19 [*Glycine max*]
35	contig13083	4-hydroxyphenylpyruvate dioxygenase [*Glycine max*]
36	contig32781	WRKY23 [*Glycine max*]
37	contig30192	WRKY54 [*Glycine max*]
38	contig05219	zinc finger (CCCH-type) family protein [*Arabidopsis thaliana*]
39	contig35898	bZIP transcription factor bZIP80 [*Glycine max*]
42	contig12172	WRKY9 [*Glycine max*]
43	contig34970	MYB transcription factor MYB57 [Glycine max]
44	contig29192	MYB transcription factor MYB183 [*Glycine max*]
45	contig29047	GAMYB-binding protein [*Glycine max*]
46	contig07725	MYB transcription factor MYB150 [*Glycine max*]
47	contig27846	MYB transcription factor MYB57 [*Glycine max*]
48	contig02140	MYB transcription factor MYB93 [*Glycine max*]
49	contig04868	flavonoid 3'-hydroxylase [*Glycine max*]
50	contig00375	flavonoid 3-O-galactosyl transferase [*Vigna mungo*]
51	contig35817	microsomal omega-6 fatty acid desaturase [*Glycine max*]
52	contig17418	omega-3 fatty acid desaturase [*Vigna unguiculata*]
53	contig08522	(iso)flavonoid glycosyltransferase [*Medicago truncatula*]
54	contig09139	enoyl-ACP reductase [*Malus *x domestica]
55	contig10732	peroxisomal fatty acid beta-oxidation multifunctional protein [*Glycine max*]
56	contig33363	beta-ketoacyl-CoA synthase family protein [*Arabidopsis thaliana*]
57	cons7	reference gene
58	contig11286	MLO8 (MILDEW RESISTANCE LOCUS O 8); calmodulin binding [*Arabidopsis thaliana*]
59	contig33974	MLO1 [*Lotus corniculatus *var. japonicus]

Almost all of the amplifications yielded single products ranging from 100 bp-150 bp showing that these are real transcripts derived from mRNA.

#### Quantitative PCR analysis of 23 common bean contigs

Of the 48 contigs whose amplification is shown in Figure 4, we randomly chose 23 contigs (Table 7) for further analysis with quantitative PCR. Randomly selected contigs were tested to determine if they were derived from RNA sequences and for their expression pattern in common bean plant parts under ambient conditions. Relative quantification of contig expression was performed by comparative ΔΔC_T _analysis from leaf, flower, pod and root tissues using leaf as a reference sample.

**Table 7:** Expression analysis of common bean contigs.

454 Contig Number	2^-ΔΔCT ^Values			Functional Annotation	Primer Sequences
			
	Flower	Pod	Root		
contig04711	3.43 ± 0.04	-2.20 ± 0.09	-0.82 ± 0.26	Alcohol dehydrogenase [*Prunus armeniaca*]	5'-ATA TGC CTC TGT CTT GGC AGG AGT-3' 5'-ACC TCG GGC AAT AGC ATT GAC TCT-3'
contig07734	2.16 ± 0.19	1.52 ± 0.07	-0.45 ± 0.1	Phosphoenolpyruvate carboxylase kinase [*Glycine max*]	5'-AGA ATG TGC GAA ACG CTG AAG ACG-3' 5'-AGG ATG GAA ACA CCG GAA GAT GGT-3'
contig08043	3.78 ± 0.18	-1.46 ± 0.17	-0.28 ± 0.18	Starch synthase III [*Phaseolus vulgaris*]	5'-AAG AAC TTG CTA GGG TGC AAG CTG-3' 5'-CTT TGC AGC TCT GTC TGC CTC AAT-3'
contig08830	-5.22 ± 0.14	6.91 ± 0.17	-1.24 ± 0.04	WRKY35 [*Glycine max*]	5'-TCA GCC TTG ACC TTG GTA TGG GAA-3' 5'-TTG CTG GTA TGA GCT TGG CTG TCA-3'
contig01300	*	-10.28 ± 0.07	1.32 ± 0.26	MADS box protein SEP3 [*Lotus corniculatus *var. japonicus]	5'-AAT TGC TCA TGC TTG GAC CTG CTG-3' 5'-TGA AGA CAT GGG ATA TGG CAG GCA-3'
contig13083	-0.69 ± 0.17	2.55 ± 0.12	1.49 ± 0.11	4-hydroxyphenylpyruvate dioxygenase [*Glycine max*]	5'-TTA TGC CAA CCT TCA CAA GCG TGC-3' 5'-TGC CCT GAT CGT CTC TGT CAA CAA-3'
contig14749	6.66 ± 0.08	0.17 ± 0.09	3.07 ± 0.27	Senescence-associated nodulin 1A [*Glycine max*]	5'-TTC TTC TTC CCT GCA CAC GAC ACT-3' 5'-TTG CTG CCC TTT CTA CGG ACA AGA-3'
contig17174	-1.41 ± 0.24	-6.58 ± 0.07	3.63 ± 0.12	Dihydroflavonol-4-reductase 2 [*Glycine max*]	5'-TGG TAG CCT CAT GCG AAC AGC ATA-3' 5'-AGG CCA GTT CGT GCA CTT AGA TGA-3'
contig14217	-1.88 ± 0.14	9.86 ± 0.1	1.15 ± 0.04	NAC domain protein NAC1 [*Phaseolus vulgaris*]	5'-TGG GTG CCC TTC CTT GAT AGA ACA-3' 5'-TGC AAC AGG GTT ACG CAC AAA TCG-3'
contig20010	6.07 ± 0.05	0.36 ± 0.08	1.34 ± 0.27	ABC transporter family protein [*Arabidopsis thaliana*]	5'-ACA ACC TTT GTT TCA GCA CGG AGC-3' 5'-GAG ACA TGG GCA ACT CAT TTG GCA-3'
contig28207	1.40 ± 0.19	-2.44 ± 0.12	-2.38 ± 0.1	Late embryogenesis abundant protein Lea14-A, putative [*Ricinus communis*]	5'-TGA CAG TCT GTT CTC CGT GTG CAT-3' 5'-TAA AGA ACC CAA ATC CGG TGC CGA-3'
contig28548	*	-1.99 ± 0.06	-0.33 ± 0.1	nodulin-26	5'-TTG GTC CAG GTC CAG CTA ACA ACA-3' 5'-CCC ATC GCC ATT GGT TTC ATC GTT-3'
contig29672	1.51 ± 0.24	3.16 ± 0.11	1.01 ± 0.07	glutathione S-transferase GST 19 [*Glycine max*]	5'-AGC TCT TCA AGG ACA CTG AGC CAA-3' 5'-AAA GGC TGT GGA TGC TGC ACT AGA-3'
contig28742	-0.84 ± 0.21	-6.13 ± 0.07	-4.19 ± 0.1	Fructokinase-like protein [*Cicer arietinum*]	5'-TGA GTA TTT GCT GAC GCG CTT CCT-3' 5'-GCA CAC CTG AAG GCA ATG GAA GTT-3'
contig28845	-0.98 ± .014	0.51 ± 0.1	-2.96 ± 0.04	NAC domain protein [*Glycine max*]	5'-TGG TGT GGT CCT GCA GAG TGT AAA-3' 5'-AAC GTC GGT GAT TGG GAG GAG AAA-3'
contig28932	4.83 ± 0.24	4.73 ± 0.07	3.81 ± 0.07	Nodule-enhanced protein phosphatase type 2C [*Lotus japonicus*]	5'-AAC GTC GGT GAT TGG GAG GAG AAA-3' 5'-CTT GCT GCT TCG CTT TGT CAC TGT-3'
contig28932	-4.04 ± 0.16	7.80 ± 0.12	-5.34 ± 0.12	nodule-enhanced protein phosphatase type 2C [*Lotus japonicus*]	5'-AAC GTC GGT GAT TGG GAG GAG AAA-3' 5'-ATC CCT CTC TCC TTC GCA GCA AAT-3'
contig30192	1.61 ± 0.22	*	0.58 ± 0.11	WRKY54 [*Glycine max*]	5'CAA CAC ACA CAT CCA AGC CCA GTT3' 5'TGG TTC TGC TGC TGC TGA TAC TGT3'
contig30958	1.17 ± 0.15	11.91 ± 0.11	3.89 ± 0.08	WRKY27 [*Glycine max*]	5'ACG GAA ACT CTG AGA GCA GCT CAA3' 5' TGC TTC CGT CCT CAC GTA AAC TCT3'
contig32565	-8.41 ± 0.15	-10.63 ± 0.11	0.90 ± 0.37	MADS transcription factor [*Glycine max*]	5'-TGC CTC ACC TAG CAA GTG TTC CTT-3' 5'-AGA TCT TGG CCC TCT AAG CAG CAA-3'
contig32665	1.26 ± 0.24	4.39 ± 0.09	0.33 ± 0.07	Transcriptional factor NAC11 [*Glycine max*]	5'-AAT GTG GTC TGA GGA GGT GGT GTT-3' 5'-ATG CTC TAA CTT CAG CGG AGG CAA-3'
contig32781	1.33 ± 0.17	1.82 ± 0.12	-2.89 ± 0.12	WRKY23 [*Glycine max*]	5'GCA TGT TGC TGT CAG GGT CAA TGT3' 5'TGG TGC TGA AGC TGA AAG TGT TGC3'
contig33251	-0.09 ± .019	-3.93 ± 0.06	-4.10 ± 0.1	Sucrose synthase [*Vigna angularis*]	5'-ACG GCT AGT TTC CTT GTG GGA GAA-3' 5'-TCT CAC ACA GCT TTC ACC CTT CCT-3'

* Data not available (inadequate replicates)

Almost all of the contigs showed expression as illustrated in Table 7. We highlight a few contigs here including contig28742 (fructose-like protein), contig2884 (NAC domain protein), contig33251 (sucrose synthase) and contig04711 (alcohol dehydrogenase) for which the expression levels were lower in flowers, pods and roots compared to leaves. Contig29672 (glutathione S-tranferase GST 19), contig28932 (nodule- enhanced protein phosphatase type C), contig30958 (WRKY27), and contig38427 (senescence-associated nodulin 1A) showed higher expression levels in flower, pod and roots compared to leaves. Expression levels of contig30958 with homology to a WRKY-27 transcription factor involved in bacterial wilt resistance [34] and contig08830 with homology to another WRKY35 transcription factor were high (119-fold) in pods compared to leaves. Expression levels of contig14749 (senescence-associated nodulin 1A), contig20010 (ABC transporter family protein) and contig38383 (fructokinase-like protein) were higher (64 fold) in flower than leaf, pods, and roots. Interestingly PCR primers designed from contig32565 showed a size difference when amplified from genomic and cDNA, and it is possible that the primers were designed from a region flanking an intron (example shown in Figure 6B).

### Identification of transcription factors

Putative common bean transcription factors (TFs) were identified by comparing *Arabidopsis *transcription factors http://plntfdb.bio.uni-potsdam.de/v3.0/ against the 454 sequencing-derived unigenes in this study [35]. A total of 2,516 unigenes coding for putative transcription factors were identified in common bean, which is similar to the 2,758 transcription factors discovered in *Arabidopsis*. However, these numbers represent about half of the transcription factors (5,671) discovered in soybean. In Table 8 we show the 16 most common transcription factor families found in common bean and corresponding TFs identified from *Arabidopsis *[35] and soybean [36].

**Table 8:** Comparison of most common transcription factor families among common bean, soybean, and *Arabidopsis *derived by screening of the *P. vulgaris *454 unigenes set against *Arabidopsis *transcription factors.

Number	TF family	Number in *P. vulgaris *unigenes	Number in *G. max*	Number in *Arabidopsis*
1	MYB	169	586	266
2	NAC	77	205	126
3	bHLH	75	390	177
4	WRKY	71	219	88
5	HB	68	279	109
6	ARF	67	75	24
7	AUX/IAA	61	97	36
8	FAR1	59	0	24
9	CCCH	58	176	85
10	PHD	50	288	59
11	Ap2/EREBP	48	405	168
12	bZIP	48	191	111
13	SET	44	0	46
14	mTERF	40	0	36
15	SNF2	32	0	45
16	MADS box	32	220	132

The largest share of common bean transcription factors (169) shows homology to the MYB super family similar to soybean (586) and *Arabidopsis thaliana *(266) which show the same abundance. This high number of MYB transcription factor identification may be due to their abundance in the genome as well as identification and characterization in model organisms. MYB genes are involved in regulation of various metabolic pathways and developmental regulation by determining cell fate and identity [37,38]. Study of these genes in common bean will help in the identification and analysis of important developmental pathways.

The second largest TF family in common bean (77) has similarity with the (NAM, ATAF1, 2 and CUC2) family as compared to 205 in soybean and 126 in *Arabidopsis thaliana *as shown in Table 8. The NAC gene family is reported to be composed of plant-specific transcription factors with a broad role in plant development (especially in lignocelluloses and cell wall development) and response to external stimuli [39]. Several NAC genes were induced by cold and dehydration in *Brassica napus *[40], by abscisic acid (ABA) and salt stress in rice [41], drought and developmental processes in chickpea [42], salinity and osmotic stress [43] and stripe rust in wheat [44].

We found 44 SET, 40 mTERF, 32 SNF2 and 59 FAR1 unigenes in our study from common bean. There are 46 SET, 36 mTERF, 45 SNF2 and 24 FAR1 transcription factors from *Arabidopsis *which are not represented in the soybean and *Medicago *transcription factor databases http://bioinfo3.noble.org/dmtr/ to date. The SET TF family is involved in methylation of lysine residues on histone tails. As of now the SET family has been found only in a few species http://plntfdb.bio.uni-potsdam.de/v3.0/. It is important to reveal the structural and functional details of these transcription factors as studies on epigenetics are expanding [45]. The SNF2 family of proteins which are DNA-dependent ATPases play an important role in chromatin remodelling complexes that are involved in epigenetic gene regulation. The mTERF family contains leucine zipper-like heptads that may be involved in mtDNA transcription and replication [46] while the FAR1 family is involved in regulation of the circadian clock in *Arabidopsis *[47].

#### Identification and analysis of nodulation-specific contigs in the unigene dataset

The 52 soybean nodulation genes discovered by Schmutz et al. [48] were compared to the common bean unigenes, using the TBLASTN algorithm. We considered as orthologs, hits with an E-value of < 1 × 10^-20 ^as per McClean et al. (22). A total of 67 hits were identified and the average E-value for these hits was 3.3 × 10^-69 ^(Table 9). Sixteen unigenes are seen to be expressed more abundantly in root tissues and this gene family will be investigated in further detail in subsequent studies.

**Table 9:** Analysis of tentative nodulation genes from 454 unigenes of common bean

unigenes	matched soybean sequences	score	E-value	454 sequencing reads			
				
				leaf	root	Pod	flower
contig34312	Glyma01g03470.1/N36a	440	3.00E-124	40	24	26	63
contig34712	Glyma01g03470.1/N36a	181	4.00E-46	1	9	12	9
contig04894	Glyma02g43860.1	499	5.00E-142	42	69	35	4
FF0DN3U02HB71T	Glyma02g43860.1	119	5.00E-29	0	1	0	0
contig27995	Glyma04g00210.1	196	1.00E-50	1	4	0	1
contig37370	Glyma05g250100.1/Nodulin-21	116	3.00E-27	6	1	1	0
contig06012	Glyma07g33070.1/SAN1B	430	2.00E-121	61	5	6	2
contig14749	Glyma07g33070.1/SAN1B	463	3.00E-131	17	1	7	4
contig38427	Glyma07g33070.1/SAN1B	607	7.00E-175	83	0	13	0
FGQI37402G5N7N	Glyma07g33070.1/SAN1B	117	1.00E-28	0	1	0	0
contig06610	Glyma07g33090.1/SAN1A	128	1.00E-30	0	25	0	0
contig31277	Glyma07g33090.1/SAN1A	409	5.00E-115	32	30	15	24
FF0DN3U02F89J3	Glyma07g33090.1/SAN1A	89	6.00E-20	0	1	0	0
contig07549	Glyma08g05370.1	328	2.00E-90	12	29	16	1
contig28119	Glyma08g05370.1	191	6.00E-49	0	8	3	2
contig30228	Glyma08g05370.1	279	2.00E-75	62	22	33	5
FF0DN3U02G2ENE	Glyma08g05370.1	117	2.00E-28	1	0	0	0
contig14951	Glyma08g12650.1/Nodulin-26a	351	1.0E-97	102	11	49	20
contig19563	Glyma08g12650.1/Nodulin-26a	119	7.00E-28	0	3	0	2
contig33328	Glyma09g33510.1/NORK	202	1.00E-53	0	56	13	0
contig05955	Glyma10g06610.1	395	3.00E-110	18	3	64	18
contig16149	Glyma10g06610.1	164	1.00E-40	19	1	45	43
FFSTDNT01DSPY6	Glyma10g06610.1	138	7.00E-35	0	1	0	0
contig05559	Glyma10g23790.1/Nod35	584	6.00E-168	54	25	13	33
contig35956	Glyma10g39450.1/Nodulin-33	251	7.00E-68	109	44	42	6
contig36020	Glyma10g39450.1/Nodulin-33	450	1.00E-127	6	27	21	195
contig14075	Glyma11g06740.1	148	2.00E-36	0	3	0	0
contig37552	Glyma11g09330.1	178	2.00E-45	5	0	53	122
FF0DN3U01DKJ7W	Glyma11g09330.2	92.4	7.00E-21	4	0	0 0	
contig29881	Glyma12g04390.1/GmNARK	428	3.00E-120	24	15	34	4
contig38136	Glyma12g04390.1/GmNARK	387	7.00E-108	205	193	377	122
FGQI37401C0XA9	Glyma12g04390.1/GmNARK	100	4.00E-23	0	0	1	0
contig06199	Glyma12g05390.1	284	5.00E-77	15	2	1	0
FFSTDNT01A6UXI	Glyma12g05390.1	111	2.00E-26	0	0	1	0
contig11587	Glyma12g28860.1	143	1.00E-34	2	0	1	0
contig33251	Glyma13g17420.1/Nod100	447	5.00E-126	8	14	27	58
contig36251	Glyma13g17420.1/Nod100	344	3.00E-95	19	10	14	21
contig36660	Glyma13g17420.1/Nod100	358	2.00E-99	11	10	27	119
FFSTDNT01BOZRN	Glyma13g17420.1/Nod100	113	2.00E-27	0	1	0	0
FFSTDNT02HOGJ9	Glyma13g17420.1/Nod100	114	2.00E-27	1	0	0	0
FGQI37402JLLRK	Glyma14g01160.1	144	1.00E-36	0	0	1	0
contig02608	Glyma14g23780.1	365	5.00E-102	61	54	83	85
contig08886	Glyma14g23780.1	292	8.00E-80	69	1	6	0
FF0DN3U02FPA3G	Glyma14g23780.1	142	5.00E-36	0	0	1	0
contig28379	Glyma14g38170.1	380	2.00E-106	95	85	73	76
contig33554	Glyma14g38170.1	338	1.00E-93	0	63	40	8
FF0DN3U01BBYR5	Glyma14g38170.1	112	5.00E-27	0	1	0	0
contig18937	Glyma15g00620.1	113	4.00E-26	3	0	0	0
contig01826	Glyma15g35070.1	170	5.00E-43	17	10	17	0
contig05700	Glyma15g35070.1	202	2.00E-52	49	34	35	10
FF0DN3U02GRTQS	Glyma15g35070.1	112	9.00E-27	0	1	0	0
contig04872	Glyma16g21620.1	699	0	13	10	26	35
contig10706	Glyma16g21620.1	229	1.00E-60	6	1	0	0
contig28435	Glyma16g21620.1	182	2.00E-46	8	28	29	1
contig06537	Glyma17g03340.1	144	6.00E-36	7	16	14	77
contig29092	Glyma17g03340.1	220	1.00E-58	6	11	3	2
contig01836	Glyma17g08110.1/Nod55-2	94.4	1.00E-20	2	1	0	18
contig06642	Glyma17g14220.1	397	3.00E-111	183	165	374	19
contig08079	Glyma17g14220.1	315	1.00E-86	3	2	4	0
FF0DN3U01DP3JJ	Glyma17g14220.1	108	1.00E-25	0	0	1	0
contig04461	Glyma17g27490.1	415	1.00E-116	6	5	19	5
contig34068	Glyma18g02230.1/N70	324	2.00E-89	75	64	19	0
contig36321	Glyma18g02230.1/N70	318	4.00E-92	16	9	16	12
FFSTDNT01DXXI7	Glyma18g02230.1/N70	99	7.00E-23	1	0	0	0
contig34962	Glyma18g14750.1	197	1.00E-93	25	16	55	41
contig08052	Glyma19g45310.1	333	6.00E-92	3	1	1	2
contig12852	Glyma19g45310.1	495	1.00E-140	9	0	5	1

## Conclusions

Genomic resources in legume crops are lacking with the exception of soybean and *Medicago *for which whole-genome sequences are now available. Since the common bean genome is relatively small compared to other legumes, there is a great potential to utilize and apply the information from common bean to other legume crops such as soybean, cowpea, mung bean, rice bean and lentils. We have partially made up for this lack of genomic information by sequencing a large number of cDNAs. In summary, we identified 59,295 common bean unigenes of which 31,664 unigenes are newly discovered sequences. Combined with existing transcriptomic and genomic sequences available for common bean, this dataset will be very useful for functional genomics research in common bean.

Comparison of the number of common bean unigene matches to other legumes shows that there may be many more legume unigenes that are yet to be discovered. Therefore, high throughput transcriptome sequencing will continue to help in identifying genes associated with biotic and abiotic stress, development of high resolution genetic maps, and automated phenotyping which will lead to crop improvement.

## Methods

### Plant materials

Common bean plants were grown in the greenhouse and leaves, flowers, and root tissues (from common bean cultivar Sierra) and pods (from common bean genotype BAT93) were collected into envelopes and frozen in liquid nitrogen for further processing.

### RNA isolation, cDNA synthesis and normalization

Total RNA was extracted from the four tissues using Plant RNA Reagents (Invitrogen; Carlsbad, CA). mRNAs (PolyA RNA) were isolated by using Oligotex Mini Kit (Qiagen; Valencia, CA). cDNA was synthesized from 500 ng of mRNA following Clontech's (Mountain View, CA) Creator SMART cDNA synthesis system using modified Oligo-dT (to make compatible with 454 GS FLX) and 5' RACE primers. The primer sequences are: CDSIII-First 454: 5' TAG AGA CCG AGG CGG CCG ACA TGT TTT GTT TTT TTT TCT TTT TTT TTT VN 3' and SMARTIV: 5' AAG CAG TGG TAT CAA CGC AGA GTG GCC ATT ACG GCC GGG 3'.

For normalization, 300 nanograms (ng) of cDNA was denatured and allowed to self-anneal in a 1 × hybridization buffer (50 mM Hepes, pH7.5 and 0.5 M NaCl) for a period of 4 hrs. Within this hybridization period, most of the abundant transcripts found their homologs to form double stranded (ds) molecules but the unique/rare transcripts and their homologs remain as single stranded (ss). After hybridization, duplex/double stranded specific Nuclease-DSN (Evrogen, Russia) was added to the reaction to degrade ds-cDNAs. Single stranded transcripts and their homologs that remained in the treated reactions were PCR amplified to make normalized ds-cDNA.

### Library preparation (DNA processing) for 454 (GSFLX) sequencing

cDNA was nebulized and size selected for an average size of 400-500 bp. 454 GSFLX specific adapters, AdapterA and AdapterB, with 10 bp MIDs in AdapterA were ligated to the cDNA ends after end-polishing reaction. The adapter ligated DNAs were then mobilized to the library preparation beads and ss-cDNAs were captured. Number of molecules of the ss-cDNAs was calculated using the concentration and average fragment length.

### emPCR, Enrichment and DNA Bead Loading

Emulsion PCR (emPCR) reactions were set up for titration run using 6 × 10^5^, 2.4 × 10^6^, 4.8 × 10^6 ^and 9.6 × 10^6 ^molecules of ss-cDNAs that corresponds to 0.5, 2, 4 and 8 copies, respectively, of the ss-cDNA per bead. Amount of ss DNA needed for the bulk run depends on the results of the titration run. In emPCR individual ss-DNA fragments were first annealed to an oligonucleotide complementary to 'B' adapter covalently bound to DNA capture beads. An emulsion was then prepared by vigorous shaking which created water-in-oil microreactors, each containing a DNA bead with attached ss-cDNA fragment and all necessary PCR reagents. This emulsion went through a thermocycling reaction that clonally amplified the attached DNA fragments to generate millions of copies of DNA on each bead. After amplification, the emulsion was broken and the beads were recovered and then washed by filtration.

The biotinylated Adapter A, also added during ss-cDNA construction, was utilized next to facilitate capture and recovery of all DNA positive beads using a streptavidin-coated magnetic bead. The capture beads without bound DNA did not bind to the streptavidin beads and were washed away. The remaining beads were then subjected to a chemical melt which denatured the bound ds-cDNA and thus separated the amplified capture beads from the magnetic beads. The mixture was magnetized again and the supernatant was recovered. This recovered supernatant contained the collection of ss-cDNA positive beads. A sequencing primer was then annealed to Adapter A and approximately 40,000 and 1,500,000 beads were loaded into a 1/16^th ^region (for titration) and into two half regions (Bulk run) respectively, of a 70 × 75 PicoTiterPlate (PTP) which contains approximately 1 million wells with an average diameter of 44 μm. This was followed by loading of the packing beads and enzyme beads. The PTP was then placed onto the GSFLX Genome Sequencer and bases (TACG) are sequentially flowed (100 cycles) across the plate. Each time a base is incorporated a chemical reaction occurs resulting in the emission of light. As chemilluminescent signal is generated it is captured by the onboard camera and processed in real time by the on-rig computer into digital images, from which DNA sequence and quality scores are generated. The raw sequences are available in SRA at NCBI under the accession number SRA028837.

### SSR analysis

To find SSRs in the data sets, the MISA program (a PERL-program written by Thomas Thiel; http://pgrc.ipk-gatersleben.de/misa) was used. The program can identify not only perfect SSRs but also compound SSRs which are interrupted by a certain number of bases. SSRs were considered to contain motifs which were between two and six nucleotides in size in this study. Dinucleotide, trinucleotide, tetranucleotide, pentanucleotide, and hexanucleotide repeats with minimum lengths of 12 bp, 15 bp, 20 bp, 25 bp and 30 bp, respectively, were considered as SSRs, as similarly defined in barley studies [49].

### Assembly and annotation of 454-reads

The adaptor sequences are identified and trim positions are changed in sff files using cross_match http://www.phrap.org, sff tools from Roche https://www.roche-applied-science.com/index.jsp and in-house Java scripts. Short sequences (less than 25 nt) were filtered and then sequences are assembled using Newbler software from Roche, using the modified sff files and default parameters. The resulting contigs and singletons that are more than 100 nt were annotated separately using BLAST [50]. The databases used were non-redundant protein database and bean ESTs from NCBI http://www.ncbi.nlm.nih.gov, *Arabidopsis *protein database from TAIR http://www.arabidopsis.org/, Soybean, *Medicago *and Lotus gene indices from DFCI http://compbio.dfci.harvard.edu/tgi/ and rice protein database from Gramene http://www.gramene.org/. In all cases the E-value cut off was 10^-5^. Top1 hits from the BLAST were parsed and used for annotation and further analysis of bean 454- contigs and singletons. The GO annotation was carried out using BLAST results against *Arabidopsis *protein sequences.

### Transcription factor analysis

We used BLAST results of bean unique sequences against *Arabidopsis *proteins, to identify bean sequences homologous to *Arabidopsis *transcription factor genes from PlnTFDB http://plntfdb.bio.uni-potsdam.de/v3.0/[35].

### Validation of expression patterns of selected unigenes

Total RNA was isolated from leaf, flower, pod and root tissues using TRIzol reagent (Invitrogen, Carlsbad, CA). Total RNA was digested with rDNAse (Ambion Inc, USA) to remove contaminated DNA. RNA concentration was measured by ND-2000 spectrophotometer (NanoDrop products, Wilmington, DE) and 10 μg of total RNA was reverse transcribed using ProtoScript^® ^M-MuLV First Strand cDNA Synthesis Kit (New England BioLabs, Beverley, MA). Removal of genomic DNA in RNA samples was further confirmed by amplifying the genomic DNA, positive cDNA, negative cDNA control with common bean molecular marker SK14 (Figure 6A) and contig 32,565 primers designed to amplify intron flanking region (Figure 6B). The 48 contigs were verified as shown in Figure 4. Randomly chosen contigs listed in Table 4 were selected for expression analysis by quantitative PCR. Concentrations of cDNA from all the tissues were equalized for reverse transcriptase and quantitative PCR experiments. The gene *cons7 *was used as an endogenous control [7,27] and leaf tissue was used as a reference sample. Real time PCR analysis was performed in 96 well format (7500 Real-Time system, Applied Biosystems, Foster City, CA) using SYBR dye. Gene expression analysis was carried out by Comparative 2^-ΔΔCT ^method [51] where ΔΔCT = (CT of contig - CT of cons7) tissue to be observed - (CT of contig.x - CT of cons7) leaf tissue.

## Authors' contributions

VK conceived and designed the research, and contributed to coordination of the analysis and experimental validation, and to the writing of the manuscript. ZL helped with experimental verification, analysis of sequence data, and contributed to writing of the manuscript. BM helped with conceiving and analysis of the research, and editing of the manuscript. JT conducted the bioinformatics analysis and contributed to the writing of the methods for 454 sequencing and the manuscript. KM helped with experimental verification and contributed to the writing of the manuscript. All authors read and approved the final manuscript.
